# High-intensity interval training (HIT) for effective and time-efficient pre-surgical exercise interventions

**DOI:** 10.1186/s13741-015-0026-8

**Published:** 2016-01-14

**Authors:** Matthew Weston, Kathryn L Weston, James M. Prentis, Chris P Snowden

**Affiliations:** Department of Sport & Exercise Sciences, School of Social Sciences, Business & Law, Teesside University, Middlesbrough, UK; Health and Social Care Institute, Teesside University, Middlesbrough, UK; Department of Perioperative and Critical Care Medicine, Freeman Hospital, Newcastle upon Tyne, UK; Institute of Cellular Medicine, The Medical School, University of Newcastle upon Tyne, Newcastle upon Tyne, UK

**Keywords:** Pre-habilitation, Patient care, Training programme, HIT, Exercise monitoring, Safety

## Abstract

The advancement of perioperative medicine is leading to greater diversity in development of pre-surgical interventions, implemented to reduce patient surgical risk and enhance post-surgical recovery. Of these interventions, the prescription of pre-operative exercise training is gathering momentum as a realistic means for enhancing patient surgical outcome. Indeed, the general benefits of exercise training have the potential to pre-operatively optimise several pre-surgical risks factors, including cardiorespiratory function, frailty and cognitive function.

Any exercise programme incorporated into the pre-operative pathway of care needs to be effective and time efficient in that any fitness gains are achievable in the limited period between the decision for surgery and operation (e.g. 4 weeks). Fortunately, there is a large volume of research describing effective and time-efficient exercise training programmes within the discipline of sports science. Accordingly, the objective of our commentary is to synthesise contemporary exercise training research, both from non-clinical and clinical populations, with the overarching aim of informing the development of effective and time-efficient pre-surgical exercise training programmes.

The development of such exercise training programmes requires the careful consideration of several key principles, namely frequency, intensity, time, type and progression of exercise. Therefore, in light of more recent evidence demonstrating the effectiveness and time efficiency of high-intensity interval training—which involves brief bouts of intense exercise interspersed with longer recovery periods—the principles of exercise training programme design will be discussed mainly in the context of such high-intensity interval training programmes. Other issues pertinent to the development, implementation and evaluation of pre-operative exercise training programmes, such as individual exercise prescription, training session monitoring and potential barriers and risks to high-intensity exercise are also discussed. The evidence presented suggests that individually prescribed and supervised high-intensity interval training programmes, encompassing a variety of exercise modes represent an effective and safe means of exercise therapy prior to surgery.

As perioperative medicine develops as a specialty, defining “fitness for surgery” will remain a priority, requiring recognition of suitable objective measures to determine levels of perioperative risk (Pearse et al. 2006; Pearse et al. 2011). The outdated perception that advanced chronological age has a “cause and effect” relationship with the increased surgical risk (Findlay 2011) has already been replaced by concentration on more specific measures of patient risk, including pre-operative frailty (Partridge et al. 2012; Makary et al. 2010), cognitive dysfunction (Bettelli 2011) and sarcopaenia (Englesbe et al. 2013).

Once developed, robust risk-stratification metrics will initially be used to inform pre-operative decision-making and the rational use of scarce post-operative resource. In addition, their role in the development of pre-operative interventions aimed at improving surgical recovery will be significant. However, such an interventional approach to pre-operative functional improvement has been limited, primarily by a lack of timely patient assessment in the context of challenging cancer and elective surgical targets.

If the perioperative medicine vision, recently presented by the Royal College of Anesthetists, is to become reality, a close synergy between well-organised patient assessment and the detection of robust objective risk markers, will lead the way towards the development of appropriately managed pre-operative interventions, applied early enough in the surgical pathway to make a difference.

The perioperative field has recently adopted the term “pre-habilitation” (Jack et al. 2011; Carli et al. 2012; Gillis et al. 2014). Loosely defined, it may refer to a group of interventions, integrated into the clinical pathway before a surgical procedure and aimed at both reducing imminent patient risk and promoting lasting beneficial effects on perioperative recovery and outcome. Presently, most of these interventions are based around simple lifestyle change including alcohol or smoking cessation, dietary measures or increasing physical activity above sedentary levels. Our focus here is on structured physical activity in the form of pre-surgical exercise interventions, the effectiveness of which has been the subject of several recent systematic reviews. Valkenet and colleagues (Valkenet et al. 2011) reported pre-operative exercise therapy to be effective for improving post-operative length of hospital stay and complication rate in patients undergoing cardiac or abdominal surgery and should therefore be considered as standard pre-operative care. More recently, a systematic review of 10 studies (1 to 12 weeks in duration) examining pre-operative aerobic exercise training in patients awaiting intra-cavity surgery reported training to be generally effective in improving physical fitness (O’Doherty et al. 2013). This finding concurs with that of Pouwels et al. (2015) who systematically reviewed five studies and reported that current evidence shows pre-operative exercise therapy in abdominal aortic aneurysm patients has potential beneficial effects. The beneficial effects of pre-operative exercise extend beyond patients undergoing abdominal or cardiac surgery as Singh et al. (2013), following a systematic review of 18 pre-surgical exercise intervention studies (median of 21 days in duration) with cancer patients, reported that pre-surgical exercise protocols containing cardiovascular and resistance exercise resulted in functional and clinical benefits that are critically important in cancer care. Therefore, despite cancer patients often receiving a diverse range of systemic cytotoxic therapies, due to a range of comorbities, that can be associated with the potential to increase the risk of exercise-related complications (Jones et al. 2010), exercise training is safe during and after cancer treatments and results in improvements in physical functioning and quality of life (Schmitz et al. 2010).

Given the increased evidence in support of pre-surgical exercise interventions, pre-operative exercise therapy is therefore likely to develop as a major component of pre-habilitation. This is not simply because exercise is known to improve cardiorespiratory function, but because of the well-documented benefits of exercise on multiple organ systems. Accepting existing cardiorespiratory function, however it is measured, as a binary concept of “fitness for surgery” is shortsighted and enforces passivity. Cardiorespiratory function in the context of an elective surgical procedure is not an end point—it is a potential starting point to therapy that may have a lasting benefit well beyond the perioperative period. The time between “decision to treat” and operation becomes an important “treatable moment”, with the opportunity to assess what part physical activity or exercise plays in the individual’s lifestyle, whether there is a general requirement to change and what support and guidance is required to motivate them to play an active role in their own pre-operative preparation. Furthermore, regaining fitness, maintaining fitness, improving fitness or indeed losing fitness could all be potential outcomes of the way clinical staff accept and promote the concept of pre-operative improvement in activity status related to their patients. Nevertheless, even where all the elements for successful behavioural modification are optimised to adapt and integrate exercise programmes into the pre-operative process, there will always be the need for appropriately designed, timely and individualized exercise programmes.

A recent article (Durrand et al. 2014) highlighted the possibility that lessons learned from exercise training within sports medicine and sports science may be transferrable to clinical practice to benefit patient outcomes. While such a notion is clearly in its infancy, we agree that the cross-fertilization of sports science with clinical practice can only help to better inform future pre-habilitative exercise interventions. Therefore, in this commentary, it is our intention to disseminate contemporary exercise intervention research from both clinical and non-clinical populations, with the ultimate aim of beginning to inform the design and prescription of effective and time-efficient exercise strategies for use prior to surgery.

## Physical activity, exercise and cardiorespiratory function

Physical activity is defined as any bodily movement produced by skeletal muscle that results in energy expenditure (Caspersen et al. 1985). Leisure time physical activity, as the name suggests, encompasses activities completed during an individual’s free time, whereas occupational physical activity is performed as part of employment (Findlay 2011; Howley 2001). Exercise is a subset of leisure time physical activity that is planned, structured and repetitive. Often, exercise is undertaken to improve and/or maintain components of physical fitness (Pearse et al. 2006; Pearse et al. 2011; Howley 2001). A subjective definition of physical fitness relates to an individual’s ability to perform everyday tasks with alertness and vigour, free of unnecessary fatigue, and with ample energy to enjoy leisure activities (Partridge et al. 2012; Makary et al. 2010; Caspersen et al. 1985). More objectively, physical fitness is a set of measureable health and performance-related attributes or characteristics that include cardiorespiratory function, muscular strength and endurance, body composition, flexibility, balance, agility, reaction time and power (Bettelli 2011; Caspersen et al. 1985; Howley 2001). Of these, cardiorespiratory function (the ability of the cardiovascular and respiratory system to supply oxygen to the working muscles during dynamic exercise) (Englesbe et al. 2013; Howley 2001) is of particular interest from a perioperative standpoint. Indeed, it has been shown that cardiorespiratory function is an independent predictor of mortality and length of hospital stay (Findlay 2011; Snowden et al. 2013) and that higher levels of pre-operative function are associated with significantly improved survival rates in individuals undergoing numerous major non-cardiac surgical procedures (Jack et al. 2011; Carli et al. 2012; Durrand et al. 2014; Snowden et al. 2013; Moyes et al. 2013; West et al. 2014; Snowden et al. 2010; Prentis et al. 2012; Older and Hall 2004). The physiological rationale relating improved cardiorespiratory function with reduced post-surgery adverse outcomes is strong—patients with adequate fitness levels will be able to better meet the neuroendocrine, metabolic and inflammatory demands of the surgery (Tew et al. 2014). Accordingly, the pre-operative period may represent a good time to engage patients in enhancing cardiorespiratory function (Durrand et al. 2014; Carli et al. 2010), with exercise training representing a plausible interventional strategy for improving surgical outcome (Gillis et al. 2014; Snowden and Minto 2015).

## Developing exercise programmes for surgery

When prescribing any exercise training programme, consideration should be given to the frequency, intensity, time, type, volume and progression (FITT-VP) principles (American College of Sports Medicine 2013). Each of these principles will be discussed in the following sections, in the context of the current UK physical activity guidelines, and exercise interventions conducted across clinical and healthy populations. Our examples will be centred on high-intensity interval training (HIT), which typically involves repeated repetitions of intense exercise, interspersed with periods of rest or low intensity active recovery (Fox et al. 1973). This is in light of accumulating evidence supporting the use of HIT to elicit substantial improvements in the cardiorespiratory fitness of patient (Weston et al. 2014a; Liou et al. 2015) and non-patient populations (Sloth et al. 2013; Gist et al. 2014; Weston et al. 2014b).

### Frequency

describes how many times per day, per week or per month exercise training takes place. In the UK “Start Active, Stay Active” report, it is recommended that all adults aged ≥19 years complete at least 150 minutes of moderate intensity activity (e.g. brisk walking) per week, in bouts of 10 minutes or more (Department of Health, Physical Activity, Health Improvement and Protection 2011). This guideline can be achieved by undertaking 30 minutes of moderate intensity activity, at least 5 days of the week. In addition, physical activity to improve strength is recommended twice weekly. While these guidelines are for physical activity, rather than exercise per se, it is widely recognised that many individuals, especially older adults (Jefferis et al. 2014), fail to meet these activity targets (Department of Health, Physical Activity, Health Improvement and Protection 2011). Although this is concerning, there is now evidence to suggest that health benefits could be achieved in a more time-efficient manner by replacing some moderate-intensity exercise with something more vigourous in nature (Department of Health, Physical Activity, Health Improvement and Protection 2011; US Department of Health and Human Services 2008). This is reflected in both the “Start Active, Stay Active” report and the American College of Sports Medicine (ACSM) exercise prescription guidance, where it is suggested that 75 minutes/three sessions a week of vigorous intensity activity can replace the 150 minutes/five sessions of moderate intensity exercise (American College of Sports Medicine 2013; Department of Health, Physical Activity, Health Improvement and Protection 2011). Given that “lack of time” remains as one of the most frequently reported barriers to regular exercise participation (Dishman 1982; Trost et al. 2002), this alternative strategy may benefit patients who cannot commit to exercising on a near daily basis, yet still want to improve their fitness in a time-efficient manner.

### Intensity

describes, in relative or absolute terms, the effort associated with the exercise. Of the FITT-VP principles, intensity is often the most important determinant of the physiological response to the exercise training (Hickson et al. 1985). As the individual response to exercise differs depending on initial fitness levels, it is recommended that exercise prescription is based on a relative measure of intensity (e.g. the energy cost of the exercise relative to the individual’s maximal capacity), rather than an absolute value (Garber et al. 2011). This is especially true for older and deconditioned populations (Howley 2001; Nelson et al. 2007).

The intensity of cardiorespiratory (endurance/aerobic) exercise can be explained practically to patients with the talk test whereby moderate exercise intensity enables a patient to comfortably hold a conversation, and high-intensity exercise precludes comfortable conversation; however, more accurate methods are available that use ratings of perceived exertion or heart-rate training zones (Jones et al. 2010). As such, relative exercise intensity can be assessed in various ways, ideally using either percentage of maximal oxygen uptake (VO_2max_), determined via a cardiopulmonary exercise test (CPET), heart rate (either percentage of maximal heart rate [HR_max_] or heart rate reserve) and individual’s perception of effort using Borg’s ratings of perceived exertion (RPE) scales (Borg 1982), with training intensities in perioperative training programmes predominately based on the latter of these measures (Jack et al. 2011). A combination of objectively and subjectively prescribed exercise intensity is likely to represent best practice here. A recent example of this was provided by Tew et al. (2014) whereby the intensity of exercise for the first training session was performed at the power output associated with ventilatory threshold determined on baseline CPET. In subsequent sessions, power output was gradually manipulated until the patient reports a perceived exertion of 6 to 7 on Borg’s CR-10 scale (Borg 1982) at the end of each work interval.

Heart rate monitoring represents a simple and reliable measure of exercise intensity and is widely considered as one of the best and most popular ways to prescribe and monitor exercise intensity (Impellizzeri et al. 2004). Often, however, patient medication can reduce the reliability of the exercise heart rate data. In situations whereby heart rate is difficult to estimate (e.g. arrhythmia or medication such as beta blockers), RPE are generally considered reliable (Eston and Williams 1988; Buckley et al. 2009) and training studies have demonstrated similar beneficial health effects when patient exercise intensity is guided by RPE compared with other more objective physiological monitoring methods (Ilarraza et al. 2004; Zanettini et al. 2012). Further, recent findings on the association between changes in RPE and heart rate during graded aerobic exercise indicate that RPE can indeed help guide exercise intensity in everyday clinical practice (Tang et al. 2015). Assuming patient familiarisation and correct researcher instructions, we recommend the use of Borg’s scales (Borg 1982), as these scales are valid for prescribing exercise intensity in clinical settings (Impellizzeri et al. 2011).

The measures described above aid categorisation of intensity of exercise training programmes. In recent guidance from the ACSM on exercise prescription (Garber et al. 2011), the term “moderate intensity” is used to describe exercise performed at either 46 to <64 % of VO_2max_, 64 to <76 % HR_max,_ or 12 to 13 on Borg’s 6 to 20 RPE scale. “Vigorous intensity” defines exercise completed at 64 to <91 % of VO_2max_, 76 to <96 % of HR_max_, or an RPE of 14 to 17; whereas exercise performed at ≥91 % of VO_2max_, ≥96 % of HR_max_, or at an RPE ≥18 is described as “near maximal” or “maximal”. It should, however, be noted that while these descriptors are helpful, they do not represent universally standardised terminology for intensity quantification within the exercise science literature. Indeed, the intensity of an exercise programme can often fall between the aforementioned ranges. An example of this is HIT, which is typically performed at ≥90 % HR_max_, therefore necessitating short interval durations (often ~30 to 60 s) and longer recovery periods. Over the last decade, there has been a surge of scientific interest on the efficacy of HIT, such that there is now accumulating evidence that, when performed over several weeks, HIT is a more effective means of improving cardiorespiratory fitness than prolonged sessions of moderate-intensity exercise (Weston et al. 2014a; Liou et al. 2015; Milanović et al. 2015). Indeed, following a meta-analysis of HIT in patients with lifestyle-induced cardiometabolic disease, Weston et al. (2014a) reported that HIT increased cardiorespiratory fitness by almost double (19.4 vs 10.3 %) that of moderate intensity continuous exercise (e.g. 50 to 60 % of VO_2max_). As such, there is a consensus for the benefit of HIT for improving cardiorespiratory fitness in patient populations (Weston et al. 2014a; Liou et al. 2015; Kessler et al. 2012; Pattyn et al. 2014).

### Time

describes the duration of the exercise session, in terms of seconds, minutes or hours. When described in this context, the UK physical activity guidelines for adults equate to 150 minutes of moderate intensity activity per week. Recently, however, it has been highlighted that by focusing solely on the 150-min recommendation, the benefits of lesser amounts of exercise could be overlooked (Sparling et al. 2015). This includes HIT, where, depending on the protocol utilised, the time spent performing exercise repetitions may only accumulate to a few minutes per session. Nonetheless, the time-efficient nature of HIT leads to greater health-enhancing benefits, including improved cardiorespiratory fitness, in less time, which increases the appeal of this form of exercise (Weston et al. 2014a; Liou et al. 2015; Milanović et al. 2015; Kessler et al. 2012; Pattyn et al. 2014).

When devising an exercise programme, the principle of time can be further broken down into time spent exercising (e.g. duration of exercise repetition), and time spent recovering (duration of rest period). This is particularly useful during interval training, where the time spent exercising and recovering can vary across trials. In the meta-analysis of Weston et al. (2014a), seven of the ten studies utilised the same type of HIT programme, which consisted of four repetitions of 4-min uphill treadmill walking/running, each interspersed with 3-min active recovery. However, to fully determine which HIT programme generates the highest benefit in terms of training response, long-term health, quality of life, and patient satisfaction, a variety of programmes should be used (Sparling et al. 2015). Furthermore, HIT actually encompasses a broad spectrum of exercise intensities and, given the pivotal role exercise intensity has on the adaptive response (Hickson et al. 1985), training sessions performed across this range of exercise intensity elicit differential adaptations (Barnes et al. 2013; Ronnestad et al. 2014). For example, Ronnestad et al. (2014) reported short HIT intervals (15 s) induced superior cardiorespiratory and power training adaptations, when compared with long HIT intervals (5 min). Low-volume HIT is a form of high-intensity interval training at the highest end of the intensity spectrum (Weston et al. 2014b). Typical low-volume HIT programmes involve 4 to 6 repetitions of 30 to 60-s maximal exercises, and these programmes elicit substantial improvements in the cardiorespiratory fitness of recreationally active and sedentary individuals (Weston et al. 2014b). While most of the low-volume HIT research to date has been performed on healthy young adults, it has been reported that 2 min of maximal intensity exercise per week for 6 weeks can be an effective strategy for counteracting age-related functional decline, reducing cardiovascular disease risk and promoting further engagement in physical activity within an elderly population (Adamson et al. 2014). The time-efficiency afforded by low-volume HIT (e.g. rapid gains in a short period of time) therefore has a clear application to perioperative care given that time-efficient exercise interventions facilitate minimal delay in surgery while also improving fitness outcomes (Durrand et al. 2014).

### Type

refers to the mode of exercise being undertaken (e.g. walking, running, cycling, dancing and resistance training). In the past, attributes such as power, balance and reaction time were described as aspects of “performance-related fitness”, such that they were almost exclusively associated with sporting performance outcomes (Howley 2001). However, it is now widely acknowledged that many basic daily activities are dependent on the ability to generate force at high velocity (Weston et al. 2014b), and therefore, power/strength training is associated with improved mobility-related outcomes, self-efficacy, satisfaction with physical function and overall life satisfaction in the elderly (Katula et al. 2008; Hruda et al. 2003). This is recognised by the inclusion of specific strength (resistance) training guidelines for adults and older adults in the current UK physical activity guidelines. It should therefore come as no surprise that, alongside cardiorespiratory fitness, pre-operative functional status can help to identify patients at higher risk of post-operative complications (Saxton and Velanovich 2011). Perioperative exercise training programmes should thus aim to improve cardiorespiratory fitness and muscle strength (Snowden and Minto 2015).

The combination of endurance training and strength training within the same training programme is referred to as “concurrent” training. While the combination of endurance and strength training is an effective means of improving strength/neuromuscular and cardiorespiratory function in healthy older adults (Cadore and Izquierdo 2013; Cadore et al. 2014; Wilhelm et al. 2014; Berryman et al. 2014; Burich et al. 2015) and patient populations, (Iepsen et al. 2015; Casla et al. 2015; Buffart et al. 2015) there is some evidence to suggest that concurrent training attenuates gains in muscle mass, strength and power compared with undertaking resistance training alone (Fyfe et al. 2014). However, the majority of literature investigating concurrent training has implemented continuous or continuous and interval endurance training protocols alongside strength training (Cantrell et al. 2014), and interference effects of endurance training are a factor of the mode, frequency and duration of the endurance training selected (Wilson et al. 2012). As such, the possibility exists that it is the total volume of endurance exercise, rather than the intensity, that may be more crucial in mediating concurrent interference (Fyfe et al. 2014; de Souza et al. 2013) and therefore, low-volume HIT protocols might confer benefit over traditional endurance training by limiting any potential volume-dependent interference effect, while also offering a similar, if not better, fitness benefit (Liou et al. 2015; Milanović et al. 2015; Fyfe et al. 2014; Cantrell et al. 2014). Ultimately, performing two types of training within the same training programme (e.g. aerobic and strength) requires an increased time commitment. Low-volume HIT can negate this, however, as simultaneous improvements aerobic fitness and strength/power have been observed following this type of training programme (Cantrell et al. 2014; Rodas et al. 2000; Zelt et al. 2014; Buckley et al. 2015); therefore, introducing a low-volume HIT programme into the perioperative care pathway has further appeal here.

The prevalent type of exercise dominating the sport and clinical HIT literature is the lower body—either cycling or treadmill walking/running (Tew et al. 2014; Weston et al. 2014a; Sloth et al. 2013; Gist et al. 2014; Weston et al. 2014b; West et al. 2015). Yet, activities of daily living require a synergy of lower and upper body fitness, and recent evidence, albeit with a small group of young to middle-aged healthy males, shows of little transfer from lower body training to upper body gains (Osawa et al. 2014). Therefore, more consideration could be given to the type of exercise prescribed for perioperative interventions. For example, Osawa and colleagues (Osawa et al. 2014) reported that a HIT programme combining upper and lower body exercise led to improved upper and lower body aerobic capacity, while also promoting muscle hypertrophy of key stabilising musculature. Heinrich et al. (2014) recently provided experimental findings that add further to the appeal of combined upper and lower body HIT. Here, the authors used “crossfit” (incorporating combined intensive upper and lower body exercises such as squats, push-ups, etc.), as the type of HIT and reported that, when compared to traditional aerobic and strength training exercises, participants spent significantly less time exercising per week, yet were able to maintain exercise enjoyment and were more likely to intend to continue (Heinrich et al. 2014). Therefore, a low-volume HIT programme delivered via exercises using a combination of upper and lower body exercises/movements would represent an attractive perioperative fitness training strategy by improving both cardiorespiratory fitness and strength/power in a time-efficient manner.

### Volume

is the product of the frequency, intensity and time principles of exercise and therefore describes the total amount of exercise performed. This principle is a major consideration when developing pre-surgical exercise interventions spanning a number of sessions, as opposed to a one-off session. Recent research on the dose-response nature of low-volume HIT has demonstrated that reducing the volume of intensive exercise does not necessarily diminish the adaptive response in healthy men (Zelt et al. 2014). In this study, the authors found that decreasing the time duration of low-volume HIT repetitions by 50 % (15-s repetitions vs 30-s repetitions) during a 4-week training intervention did not diminish adaptations in cardiorespiratory fitness or muscular power (Zelt et al. 2014). Establishing a minimal effective amount of HIT (Metcalfe et al. 2012; Gillen et al. 2014) therefore represents an attractive line of investigation for researchers, as does examination of the minimal amount of exercise required to maintain prior HIT-induced fitness gains (Tew et al. 2014; Macpherson and Weston 2015). More work examining the “optimal” amount of HIT for firstly developing and secondly maintaining perioperative fitness, in case of delayed surgery, would be welcomed as such research would clearly be for patient benefit.

### Progression

relates to how the prescription of exercise training advances over time. This can be achieved by increasing the frequency, intensity or duration of an exercise programme, though it is recommended that only one element should be changed at a time (American College of Sports Medicine 2013). Prior to making any adjustments, however, it should first be ensured that each aspect of the programme is being measured accurately. Assessing the frequency of exercise sessions can be recorded through a session attendance register, or an individual diary. Ascertaining whether individuals are consistently performing at the correct exercise intensity throughout their programme relates to the concept of intervention fidelity—the demonstration that an experimental manipulation (in this case, an exercise programme) has been implemented as intended, in a comparable manner to all participants (Dumas et al. 2001). An assessment of fidelity is integral to the internal validity of intervention-based trials (Bellg et al. 2004; Horner et al. 2006), by providing greater insight into the true relationship between exposure (the exercise programme) and outcome (Taylor et al. 2015). By measuring and reporting the exercise intensity on a session-by-session basis (e.g. within-session RPE scores), along with physiological changes determined via mid- and post-intervention assessments (e.g. a CPET) researchers are able to ascertain whether the fidelity of the intervention has been upheld for all participants (Weston et al. 2014b). Further, relative measures of exercise intensity such as RPE scores provide a practical and valid means of ensuring training progression is inherent within programmes. While responsive training, whereby training intensities are re-defined and re-prescribed following a mid-programme assessment of exercise capacity (West et al. 2015), can also provide information to aid training progression, this is at greater fiscal and time cost. Such assessments can also disrupt the frequency of an exercise training programme, since the exercise capacity test itself will count as an exercise session. As such, it is recommended that assessments be accounted for when designing the training programme.

Running and cycling are physiological tasks that elicit dissimilar dimensions of central and peripheral fatigue (Millet et al. 2009); therefore, from a practical perspective, it is important that CPET informed training sessions are performed on the same mode of exercise equipment as the test; otherwise, the intensity may no longer be in the prescribed domain. It is also imperative that the effectiveness of exercise programmes devised to improve both aerobic fitness and strength/power is assessed with appropriate tests that permit the detection of change in these disparate fitness components. For example, studies showing strong similarities between training and testing routines are more likely to show training improvements (Buchheit 2012), and with this in mind, we recommend the inclusion of functional fitness tests to assess the transference of intervention effects to the activities of daily living. Therefore, along with, or potentially in replacement of traditional laboratory tests of aerobic fitness (e.g. VO_2max_, anaerobic threshold) and strength/power (e.g. isokinetic dynamometry, 1 repetition maximum), such tests as the 6-min walk test (Bellet et al. 2012), 30-s chair stand test (Jones et al. 1999) and the 8-foot up-and-go test (Rose et al. 2002) have appeal for the assessment of fitness changes in the perioperative setting.

## Other considerations

At the individual session level, consideration should be given to the training principles of intensity, time and type. When developing an exercise programme spanning a number of weeks or months (less likely in the pre-surgical context), however, thought must also be given to how often the sessions will take place, how they will progress over time and the overall volume of exercise prescribed. Consideration should also be given to the perceived barriers to exercise participation that patients may face. For example, opponents of HIT, and in particular low-volume HIT, often highlight that the intense physical effort required and associated fatigue may be detrimental to motivation (Sparling et al. 2015). This claim, however, has not been evidenced in the literature as adherence rates to clinical and non-clinical HIT programmes have been high, albeit in relatively small samples sizes, and often display better adherence than “traditional” moderate-intensity exercise programmes (Wisloff et al. 2007; Currie et al. 2015). Further discussion surrounding this topic can be found elsewhere (Biddle and Batterham 2015).

It has also been suggested that performing HIT may increase injury risk and medical complications (Lunt et al. 2014). While these are important issues to be aware of, risks can be greatly minimized through proper consideration of the FITT-VP principles from the programme onset, and ensuring that the exerciser performs an adequate warm-up. Ensuring that exercise intensity is set relative to the individual’s capacity, as opposed to a standardised value, can also prevent stagnation, over-exertion and the risks associated with this. Furthermore, following a recent systematic review of HIT in patients with cardiovascular and metabolic diseases, Levinger et al. (2015) reported that in all studies, the rate of adverse responses was low, and HIT sessions were well tolerated overall across all patients. We concur with the safety advice provided by these authors whereby patients undertaking HIT should be clinically stable, have had recent exposure to at least regular moderate intensity exercise, undertake the training in facilities that have both the equipment and the expertise to handle adverse responses and have appropriate supervision and monitoring during and after the exercise session.

Perioperative exercise training represents a credible means for improving surgical outcome. In this commentary, we have attempted to combine contemporary exercise training research from both exercise science and clinical science, to inform on key issues related to exercise training programme design, namely frequency, intensity, time, exercise type, volume and training progression. Collectively, the evidence presented on the FITT-VP principles with regard to HIT support its use as a promising perioperative strategy for enhancing cardiorespiratory fitness. We are not suggesting that HIT should be used as a replacement for all other forms of exercise and physical activity; however, given the need for pre-surgery exercise interventions to be both effective and time efficient, we believe that carefully designed and supervised HIT programmes targeting the upper and lower body and tailored to the individual, represent a valuable addition to the perioperative pathway of care.

## Abbreviations

ACSM: American College of Sports Medicine
CPET: cardiopulmonary exercise test
FITT-VP: frequency, intensity, time, type, volume and progression
HIT: high-intensity interval training
HR_max_: maximal heart rate
RPE: ratings of perceived exertion
VO2_max_: maximal oxygen uptake
